# Can Adenosine Fight COVID-19 Acute Respiratory Distress Syndrome?

**DOI:** 10.3390/jcm9093045

**Published:** 2020-09-21

**Authors:** Carmela Falcone, Massimo Caracciolo, Pierpaolo Correale, Sebastiano Macheda, Eugenio Giuseppe Vadalà, Stefano La Scala, Marco Tescione, Roberta Danieli, Anna Ferrarelli, Maria Grazia Tarsitano, Lorenzo Romano, Antonino De Lorenzo

**Affiliations:** 1 Unit of Radiology, Grande Ospedale Metropolitano Bianchi Melacrino Morelli, 89124 Reggio Calabria, Italy; milafalcone@gmail.com (C.F.); anferrarelli@yahoo.it (A.F.); 2 Unit of Intensive Postoperative Therapy, Grande Ospedale Metropolitano Bianchi Melacrino Morelli, 89124 Reggio Calabria, Italy; maxcar04@libero.it; 3 Medical Oncology Unit, Grande Ospedale Metropolitano Bianchi Melacrino Morelli, 89124 Reggio Calabria, Italy; correalep@yahoo.it; 4 Unit of Intensive Care Medicine and Anesthesia, Grande Ospedale Metropolitano Bianchi Melacrino Morelli, 89124 Reggio Calabria, Italy; nucciomacheda58@gmail.com (S.M.); eugvad@yahoo.it (E.G.V.); stelascala66@gmail.com (S.L.S.); marcotescione.mt@gmail.com (M.T.); 5 Department of Human Sciences and Promotion of the Quality of Life, University San Raffaele, 00166 Rome, Italy; roberta.danieli@uniroma5.it; 6 Department of Experimental Medicine, University of Rome Sapienza, 00161 Rome, Italy; mariagrazia.tarsitano@uniroma1.it; 7 School of Specialization in Food Science, University of Rome Tor Vergata, 00133 Rome, Italy; 8 Section of Clinical Nutrition and Nutrigenomics, Department of Biomedicine and Prevention, University of Rome Tor Vergata, 00133 Rome, Italy; delorenzo@uniroma2.it

**Keywords:** COVID-19, ARDS, adenosine, CT-scan, cytokines storm

## Abstract

Coronavirus disease 2019 (COVID-19) patients can develop interstitial pneumonia, which, in turn, can evolve into acute respiratory distress syndrome (ARDS). This is accompanied by an inflammatory cytokine storm. severe acute respiratory syndrome coronavirus type 2 (SARS-CoV-2) has proteins capable of promoting the cytokine storm, especially in patients with comorbidities, including obesity. Since currently no resolutive therapy for ARDS has been found and given the scientific literature regarding the use of adenosine, its application has been hypothesized. Through its receptors, adenosine is able to inhibit the acute inflammatory process, increase the protection capacity of the epithelial barrier, and reduce the damage due to an overactivation of the immune system, such as that occurring in cytokine storms. These features are known in ischemia/reperfusion models and could also be exploited in acute lung injury with hypoxia. Considering these hypotheses, a COVID-19 patient with unresponsive respiratory failure was treated with adenosine for compassionate use. The results showed a rapid improvement of clinical conditions, with negativity of SARS-CoV2 detection.

## 1. Introduction

The current coronavirus disease 2019 (COVID-19) outbreak has been declared a pandemic by the World Health Organization (WHO), reporting more than 13 million new cases worldwide with 572,539 related deaths [1]. Part of these patients develops interstitial pneumonia, which can, in turn, evolve into acute respiratory distress syndrome (ARDS). ARDS patients require active hyperoxic ventilation, with mainly fatal outcomes [2]. The pathogenesis of COVID-19-related lung injury is still unclear and under study, but the cytokine storm is a severe event with a poor prognosis [2]. For this reason, various drugs have been tested to block such storm. Dramatically, over 50% of COVID-19 patients needed respiratory support. Studies have shown that some lung complications are due to iatrogenic injury [3]. A previous preclinical study using an animal model [3] observed that oxygenation inhibits the physiological mechanism of protection of the tissues, and this could aggravate ARDS. For this reason, the role of the puraminergic system in preventing respiratory complications in COVID-19 has been investigated. In fact, respiratory support to the COVID-19 patient could contribute to the inhibition of the adenosine pathway mediated by the A2A receptor (A2AR). This pathway involves shutting down the acute inflammatory process in cases of hypoxia.

The following review and clinical case aim to clarify the molecular mechanisms involved in the possible use of inhaled adenosine as a therapy to hinder the cytokine storm in COVID-19.

## 2. COVID19 Pathogenesis, Mitochondrial Dynamism and Immune Response

The severe acute respiratory syndrome coronavirus type 2 (SARS-CoV-2) is the pathogen responsible for the COVID-19 pandemic [4]. Parallel to the SARS-CoV-2 virology, COVID-19 pathophysiological immune processes have been investigated to identify effective therapies. SARS-CoV-2 can infect the lower respiratory tracts causing potentially fatal pneumonia [5]. After infection, symptoms appear on average within 11.5 days [6]; the viral peak is reached 6 days after symptoms [7], and COVID-19 progressive worsening in ARDS may occur within 8–9 days [7].

The host’s response to the infection determines the severity of the inflammation, which can lead to the cytokine storm. SARS-CoV-2, using angiotensin converting enzyme 2 (ACE2), primarily infects the lung epithelium and promotes inflammation and vascular permeability of the airways [8]. Infection and viral cytopathic damage lead to death of the pulmonary epithelium and the recruitment of macrophages, monocytes’ release of cytokines, and thus the adaptive immune responses of T and B cells [9]. This reaction resolves the infection in most cases. On the other hand, a dysfunctional immune response causes severe pulmonary and systemic pathology [4]. There are numerous factors that influence the host response, and mitochondria are involved in this process. Mitochondria are key organelles involved in energy metabolism, aging, apoptosis, and the innate immune response [10].

The nature of mitochondria is dynamic and not static. In fact, in order to better preserve their functions, these organelles activate mechanisms of fission, mitophagy, and fusion. Those processes are sensitive to cellular physiological and pathological changes and allow a response to stress. Mitochondrial dynamism has a central role in the signaling hub of the innate immune response, effective in contrasting viral pathogenesis [11]. In short, the cyclic and dynamic mitochondrial process eliminates the damaged parts of the mitochondria, recycles the damaged structures, and merges mitochondria matters [12]. Fission is coordinated by dynamin-related proteins (DRP), mainly by DRP-1. Following polarization, as a result of mitochondrial damage, DRP-1 oligomerizes as a ring and causes the splitting of mitochondrial membranes [13]. Subsequently, the parkin-dependent mitophagy process of the damaged portion of mitochondria is facilitated by selective phosphatase and tensin homolog-induced kinase 1 (PINK-1) signaling [14]. Finally, the fusion process of the repaired parts takes place thanks to the mito-fusins present on the mitochondrial membrane [15].

Some molecular patterns associated with viruses are recognized by the retinoic acid-inducible gene 1 (RIG-I) and its RIG-I-like receptors (RLRs). By recognizing the double helix RNA, they activate the mitochondrial-associated antiviral signaling protein (MAVS) [16]. Mitochondrial fusion and fission activate or attenuate this pathway, with the aim of amplifying the production of interferons and initiating the antiviral immune response. By distorting the mitochondrial dynamism, viruses can prevent the correct production of MAVS [16]. MAVS, binding to caspase recruitment domains (CARDs), promote nuclear factor kappa-light-chain-enhancer of activated B cells (NFkB) activation and interferon induction [11].

In addition, the viral strategy in modulating the mitochondrial function provides [11] the following: impaired calcium regulation; increased oxidative stress; regulation of membrane mitochondrial potential; apoptosis. The human coronavirus (hCoV) possesses accessory proteins, open reading frame (ORF) 3a, 8a, 6, 7a and 8b, which promote the pathogenesis mechanism. ORF3a and ORF8a trigger apoptosis; ORF3b promotes the release of different cytokines and chemokines; ORF6 inhibits the production of interferon (INF); and ORF7a promotes transcription mediated by NFkB [10]. Lastly, the role of ORF9b was identified. This protein is responsible for inhibiting MAVS, limiting the innate immune response [17]. In detail, ORF9b promotes the degradation of DRP-1, thus inhibiting mitochondrial fission and MAVS signaling. This process determines cell survival during viral replication [17]. MAVS signaling including the TNF receptor associated factor (TRAF)3/TRAF6 signalosome is downregulated, resulting in decreased INF release and lack of innate immune response [17].

In SARS-CoV-2, several accessory proteins have been identified in common with SARS-CoV [18]. In particular, the same ORF9b sequence as in SARS-CoV was detected in a different position of the viral genome [17,19]. As clinically observed, it would seem that the current virus has all the pathogenetic characteristics to interfere deeply with the innate immune response, to determine a delay in the response, to reduce the production of INF and, at the same time, to activate the polymorphonuclear leukocytes (PMNs) response and subsequent cytokine storm.

In conclusion, the repair mechanism of mitochondria is compromised and instead of leading to cell apoptosis, the virus promotes cell survival and its replication.

## 3. Cytokine Storm

The high mortality in COVID-19 patients has been closely related to the cytokine storm. This is an excessive immune response, with a complex pathogenesis, determining rapid progression and high mortality from the disease [2]. Curiously, numerous patients who quickly died of ARDS did not experience severe symptoms in the early stages of the disease but only the common mild ones.

The cytokine storm is among the causes of ARDS and multi organ failure (MOF) and is detected in COVID-19 patients [2]. It is clear that in critically ill COVID-19 patients, the pathogenesis is promoted by a specific proinflammatory response. In vitro experiments have shown that in the early stage of a SARS-CoV infection, infected cells (respiratory epithelial cells, dendritic cells, and macrophages) delay the release of cytokines and chemokines [20,21,22]. Subsequently, antiviral molecules belonging to the interferon family are secreted at low concentrations, while proinflammatory cytokines (IL-1β, IL-6, tumor necrosis factor (TNF)-α, and chemokines are widely released [20,21,22].

In previous coronavirus epidemics, high levels of cytokines and chemokines in infected patients have been observed. In particular, a high number of neutrophils and monocytes in the bloodstream and lungs have been detected [23,24,25,26]. At the same time, the delay in interferon secretion results in a reduced and altered antiviral response. Interferons represent the molecular key of the human body defense against virus infection [27].

Afterwards, the high level of cytokines and chemokines determines the infiltration of numerous inflammatory cells into the lung tissue, such as lymphocytes, monocytes, and macrophages. A greater deregulation has already been observed in older primates and other animal models [28].

In hCoV infection, the ARDS determinants are the delay in the antiviral response, the secretion of cytokines, chemokines, granulocyte-macrophage colony-stimulating factor, reactive oxygen species, chemokines, and cell apoptosis products [29]. All this causes apoptosis of the endothelium and pulmonary epithelium, destruction of the respiratory barrier, and vascular leakage with alveolar edema [30].

**Figure 1:**
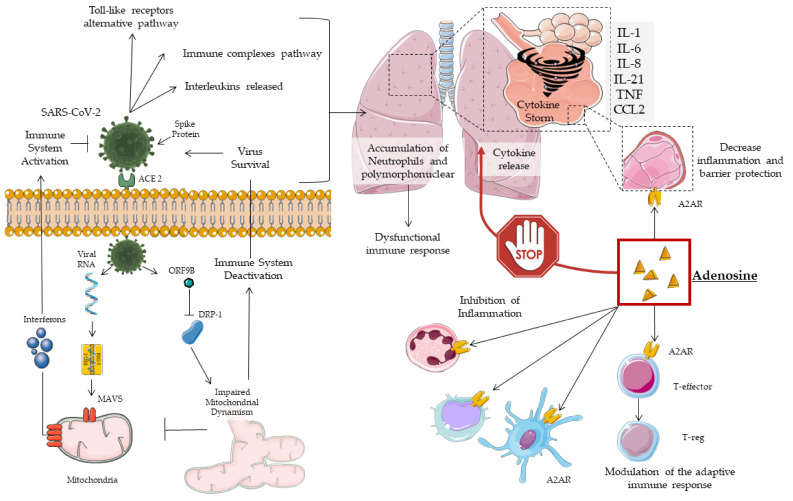
Mechanisms of adenosine action on COVID-19.

A positive correlation between cytokine levels and disease severity was also found in COVID-19 patients, with non-specific recruitment of inflammatory cells. In addition to the pro-inflammatory molecules described so far, COVID-19 patients also showed a reduced presence of anti-inflammatory cytokines, with an imbalance in favor of the former [17] (Figure 1).

In COVID-19, obesity and related-comorbidities increase the risk of intensive care unit (ICU) hospitalization and death. This occurs due to a rise in inflammation status, impaired immune response, and respiratory dysfunction [30,31,32,33,34,35]. Obesity has been shown to be an independent risk factor in young males [36]. Metabolic syndrome, frequent in obesity, promotes the cytokine storm [35,36]. Diabetes mellitus, due to hyperglycemia, impairs the immune response and acts to ACE2 glycosylation [37,38], favoring the infection and pathogenesis of SARS-CoV-2 [39].

In summary, obesity and its comorbidities increase the patient’s inflammatory status, resulting in a higher inflammation set-point and susceptibility to infection. This phenomenon has been called immuno-innate memory and is attributable to epigenetic changes that we know to be influenced by the environment, lifestyle, and poor nutrition [40]. In COVID-19, all these factors predispose to an immune dysfunction that leads to increased respiratory complications. Hence, the importance of anti-inflammatory therapy, such as adenosine, as well as antivirals is highlighted.

### Current COVID-19 and Cytokine Storm Therapeutic Strategies

Several therapies, already in use, have been adopted to combat COVID-19. Basically, they can be grouped into two large groups: antiviral therapy and immunotherapy [41]. First, glucocorticoids are administered in the more critical or early stages of the cytokine storm to prevent organ damage. On the other hand, too early administration can inhibit the immune response, with viral proliferation, and at high doses, it can delay viral clearance [42,43]. Among the anti-inflammatory drugs, hydroxychloroquine (HCQ) was approved in emergency in March. It was widely used because it inhibits the production and release of TNF and IL-6, which has useful effects in COVID-19 patients [44,45]. However, the action on viral proliferation is not clear and is currently related to the ability of blocking lysosomal degradation and autophagy [46]. A recent study found that administration of 200 mg twice daily reduces hospital mortality by 30%. However, the authors concluded that this finding needs to be transferred with caution to clinical practice [47]. In addition, the combination of HCQ with protease inhibitors lopinavir/ritonavir and, later, the more recent darunavir and remdesivir [48] were investigated. For antivirals, controversial and not conclusive results have been observed [49,50].

The immune-based therapies, targeting factors involved in the cytokine storm, including monoclonal antibodies such as anakinra, an interleukin 1 receptor antagonist [51], canakinumab IL-1β, a selective inhibitor used in patients with ARDS [52], and tocilizumab IL6 inhibitors (TCZs) have been studied in several clinical trials [49,50,51,52,53]. In particular, the serum level of IL-6 is significantly increased in severely ill COVID-19 patients, inducing the administration of TCZ in patients. Recently, a paper from the Italian Medicines Agency (AIFA) reported that early administration of TCZ in COVID-19 patients did not lead to relevant clinical benefits [54]. The potential effects of TCZ in selected patient subgroups remains to be elucidated [4]. In addition, other therapies, such as Janus kinase inhibitors (JAK) -1/2 (baricitinib and ruxolitinib), have been tested, but, to date, there are insufficient data to support their use [55]. Of interest was the study with acalabrutinib, a Brut kinase inhibitor, that observed the ability to reduce the need for mechanical ventilation in 25% of patients [3]. Currently, without an approved therapy to COVID-19 and given the recognized anti-inflammatory action of adenosine, its use could be hypothesized. Its known ability to inhibit inflammation could act in acute lung injury as a local and systemic anti-inflammatory by stemming the cytokine storm [55,56,57].

## 4. Adenosine

Adenosine is a molecule belonging to the oldest signaling systems, probably linked to biological function [55]. This purine has a half-life in vivo of 1.5 s and is generated by the hydrolysis of polymers. Its messenger activity is carried out in the extracellular space, which the purines reach thanks to transporters, hemichannels, damaged cell membranes, or degranulation as adenosine diphosphate (ADP) [58]. In the extracellular space, adenosine triphosphate (ATP) and ADP are converted to adenosine monophosphate (AMP) by the exonuclease enzyme cluster of differentiation (CD) [37]. Subsequently, AMP is hydrolyzed into adenosine by the enzyme CD73 [59]. The bioavailability of adenosine is limited over time, and this is due to the nucleoside transport systems [60], diffusion by gradient, metabolization to inositol through adenosine deaminase [61], and phosphorylation in AMP via adenosine kinase [62]. In addition, adenosine has four receptor subtypes belonging to the G-protein superfamily. The short half-life of adenosine may be sufficient given the rapid recruitment of second messengers and prolonged activation of the pathways involved [63].

Knowledge about the role of adenosine in attenuating and modulating an excessive inflammatory response is emerging. In experimental ischemia/reperfusion models, adenosine and its agonists have been shown to block infiltration, trafficking, activation of PMNs and production of superoxides, with mitigation of reperfusion damage [64]. By the adenosine signaling, platelets, endothelial cells, macrophages, T cells, and mast cells are also modulated for an anti-inflammatory action [65]. Given these properties, adenosine could be used to treat acute lung injury (ALI) and ARDS [66,67]. In fact, the response of PMNs to adenosine and the presence of receptors for it on the human lung reinforce this hypothesis [68]. In many animal models of ALI and ARDS, adenosine or specific agonists have shown the ability to reduce inflammation, regulate endothelial integrity, and balance lung fluids [68,69,70].

Finally, an increase in endogenous adenosine production was observed in response to mechanical ventilation damage. Some authors have speculated that endogenous production might mitigate lung damage [70], while others have pointed out that oxygenation reduces the physiological mechanism of tissue protection in response to exogenous adenosine [3]. These preclinical studies analyzed different experimental models and require further confirmation. In COVID-19 patients requiring respiratory support, adenosine treatment should be carefully considered. Furthermore, adenosine diffused through the lungs could reach severely damaged areas, without losing its effectiveness in hypoxic conditions.

### 4.1. Subtypes of Adenosine Receptors (AR)

Adenosine receptor subtype A1 is mainly located in the central nervous system, and its impact is still poorly understood [68]. It has been observed that its activation is not protective against inflammation, damage from reperfusion, or capillary pulmonary filtration [71]. The chronotropic action of adenosine, namely the slowing of the heart rate, is mediated by subtype A1 [57]. Indeed, efforts have focused on finding a selective antagonist/agonist for this receptor [56]. 

The A2A subtype is the most common in the human body [72], and its expression is increased in case of damage, especially in macrophages [73]. In SARS-Cov2 the infection of the alveolar epithelium immediately involves the nearby endothelial cells and alveolar macrophages. A release of pro-inflammatory molecules is triggered, with an inflammation affecting the alveolar cellular architecture. This attracts monocytes, macrophages, and T cells to the infection site, promoting an increase in inflammation. In some patients, a dysfunctional immune response leads to an accumulation of immune cells in the lungs and the death of lung tissue due to an overproduction of pro-inflammatory molecules. The conditions of multi-organ damage mediated by the cytokine storm and the inability of the host to prevent the spread of the virus are still configured at a systemic level [4]. Therefore, adenosine can help control the inflammatory response and, in turn, increase the response of the host against the virus. Antiviral and regulatory therapies for a dysfunctional immune system could act in synergy by blocking replication and reducing clinical severity [4]. The cellular responses are different: coronary vasodilation, platelet aggregation inhibition, inhibitory modulation, non-redundant [74], acute immune response against T cells, monocyte, macrophages, PMNs, and dendritic cells [75]. The role of A2A in protecting lung integrity from the acute inflammatory process is evident. In fact, the activation determines the suppression of the immune response, the reduction of microvascular permeability and capillary filtration of the lung, preservation of fluid homeostasis, increased clearance of alveolar fluids, and inhibition of trans epithelial leukocyte migration [3,76]. In acute inflammation, the evaluation of alveolar bronchial lavage has shown that the activation of A2A determines a reduction in the secretion of IL-6, TNF-α, and chemokine (C-X-C motif) ligand 3 (CXCL1-3) [77]. The A2B subtype is widespread in the body, strongly expressed in the vascular compartment and macrophages, but with a lower affinity. More generally, it has been observed that adenosine is able to promote the processes of angiogenesis and vasculogenesis through the stimulation of VEGF, especially following damage to the endothelium. Furthermore, in conditions of stress, adenosine is an effective vasodilator, able to regulate the vascular traffic of leukocytes, and plays a role in protecting the lung from mechanical ventilation [78,79]. The damage related to mechanical ventilation has been attributed to an excess of pressure or volume, which is responsible for the “shear stress” to the lung parenchyma. This results in an inflammatory cascade against, in which adenosine could play a protective role. 

Finally, the adenosine receptor subtype A3B is ubiquitous, but specifically located in the lung, liver, and eye [72]. Animal models have shown a pro-inflammatory capacity. In humans, only the control of the degranulation of PMNs is clear [80]. Moreover, its activation regulates the production of aqueous humor, and selective activation is being tested in dry eye syndrome [81].

### 4.2. Adenosine in the Treatment of Lung Injury

Currently, adenosine finds its use in the treatment of paroxysmal supraventricular tachycardias, also in the pediatric population [82]. In addition, therapeutic strategies are being studied involving the use of adenosine and drugs that interact with its metabolism for the treatment of cancer-mediated immunosuppression and other pathologies with a dysregulation of the inflammatory response [83,84]. ATP released following an injury performs a diversified signaling function. In fact, its receptors are present in different cells involved in inflammation [59]. In lung damage, the role of ATP is controversial. Its action is also expressed in the production of the surfactant and in the regulation of lung microbiome [85,86].

The exonucleases are central in the anti-inflammatory response, to produce adenosine and to activate regulatory T cells (T Regs) for CD73 [87]. In lung inflammation, adenosine promotes the cellular response to hypoxia [88] and the reduction of extravasation of proteins and cytokines in the alveolus with decreased infiltrated neutrophils [89]. A powerful synergy between peroxisome proliferator-activated receptor γ (PPARγ) and adenosine has been observed. They upregulate one another in the expression of proteins involved in the attenuation of edema, improvement of gas exchange, and lung function [90]. Furthermore, adenosine is favored by the presence of CD73 on the T Regs activated through A2A, acts as modulator of the immune response, and assists in the resolution of the acute phase [91]. A2A expressed in PMNs reduces their traffic, infiltration, and activation, attenuating the inflammatory lung response [89]. As already discussed, most of the anti-inflammatory functions of adenosine are promoted by the activation of A2A. However, during hypoxia and mechanical ventilation, the excess production of adenosine also activates A2B [92]. In particular, a dose-dependent response of pulmonary permeability [93], involving the reduction of interleukins, TNF-α, and chemokines with attenuation of the inflammatory cellular response [94], has been observed. A2B has a lower affinity for adenosine. Still, in pathological conditions of hypoxia and extensive inflammation, it is activated to counteract the inflammatory response and prevent further damage.

To date, the standard treatment for ARDS remains respiratory support therapy such as ventilation with oxygen at high concentrations. However, iatrogenic hyperoxygenation is likely to weaken the anti-inflammatory action of adenosine. In fact, the signaling of adenosine and of the A2A receptor is activated in conditions of hypoxia, as in the ischemia reperfusion model. Under severe conditions and dysregulated inflammation, such as in COVD-19, hyperoxygenation may exacerbate lung damage. This phenomenon has been studied in the animal model. It has been highlighted that during hyperoxygenation, the enhancement of adenosine signaling protects the lung tissue, and, contrarily, a weakening causes greater damage [3].

## 5. Clinical Case

Hereafter, we describe the clinical case of a patient with COVID-19 hospitalized at the Great Metropolitan Bianchi Melacrino Morelli (GOM) Hospital in Reggio Calabria, Italy. The patient, given the lack of clinical improvement, was provided charitable therapy with adenosine via the inhalation route. The patient was monitored daily and subjected to instrumental tests. A computed tomography (CT) scan was performed at baseline and after treatment, and results were analyzed by the same radiologist. The detection of SARS-Cov-2 in the upper or lower upper or lower respiratory tract was performed at admission, at baseline, 48 h, 120 h, and 15 days after the beginning of treatment. The detection of SARS-Cov-2 was evaluated by the laboratory of Microbiology& Virology of the GOM. Upper (nasopharyngeal swabs) and lower (broncho-alveolar lavages, broncho-aspirates, and tracheal aspirates) respiratory tract specimens, were collected using Copan Universal Transport Medium (UTM-RT®) System or a sterile container at 4°C and processed within 24 h. RNA-COVID-19 was evaluated using an Allplex 2019-nCoV Assay that identifies three different target genes, namely E (envelope), RdRp (RNA-dependent RNA polymerase), and N (nucleoprotein gene), according to the international recommended guidelines by the World Health Organization. The test assay was performed following the manufacturer’s instructions. According to the interpretation criteria, detection of only one of multiple genes has been interpreted as a COVID-19 positive result. 

Inhalatory adenosine was nebulized and dispensed by an Aerogen USB Controller linked to a high flux device with 21% FiO_2_, a flow of 60 L/min in 5 min. Adenosine dosage of 9 mg every 12 h in the first 24 h and subsequently, every 24 days for 4 consecutive days was administered [95]. The inhaled adenosine dose was extrapolated from preclinical studies in mice [96,97,98,99] as well as from clinical studies using adenosine as an aerosol formulation, showing dose limiting efficacy over 10 mg and no adverse events in normal individuals and patients with non-asthmatic disease [97,98,99,100,101]. The off-label treatment and patient monitoring was approved by the Hospital Safety Team and by the Ethical Committee of South Calabria. Patient’s privacy and sensitive data were appropriately protected.

On 31 March 2020, a 58-year-old man with normal weight for BMI (24.8 kg/m^2^) was hospitalized for COVID-19-related acute respiratory failure with hypoxemia. Of note in his past medical history, obstructive sleep apnea syndrome and previous surgery for the removal of an epidermoid cyst of the left ponto-cerebellar corner were reported. The chest CT examination on March 31 was performed in basal conditions but was significantly limited by the patient’s inability to maintain adequate respiratory kinetics. Consolidating areas of the lungs and visceral pleural thickening with a tendency to confluence to the lower lobes were observed. Furthermore, ground-glass opacity and increased density, the presence of consolidating components that largely affect the upper lobes, with associated diffuse reticular interstitial thickening were reported. Also, ground-glass areas with blurred contours in correspondence with the antero-medial segment of the right upper lobe (RUL), presence of ground-glass lesions in the lower lobes, and presence of pseudonodular-looking fibrosclerotic lesions arranged in clusters bilaterally in the lower lobes were found. Again, pleuroparenchymal fibrosis of a probable disventilatory nature was present in the posterior basal segments of both lower lobes. Mediastinal lymphadenomegalies were not evident. From April 1, 2020, the patient was treated with low molecular weight heparin 4000 IU twice daily until discharge. Moreoever, hydroxychlororoquine 200 mg twice daily, azithromycin once daily, and oxygen therapy with Ventimask were administered until April 13. After 2 weeks following admission, due to the absence of improvement in respiratory failure with an average PaO_2_/FiO_2_ value <250, the patient was offered charitable treatment. The patient was informed and received off-label treatment with inhalatory adenosine. After 5 days since the first administration, a rapid increase in PaO2/FiO2 was observed, up to an average value >400, with improvement of biochemical parameters (Table 1), respiratory failure, and interruption of respiratory support. Furthermore, with regard to the detection of SARS-CoV-2 on nasopharyngeal swabs, a positive result was observed only for the N gene on April 17. In the following detections, carried out as per protocol every 2 days, it was always found negative.

**Figure 2:**
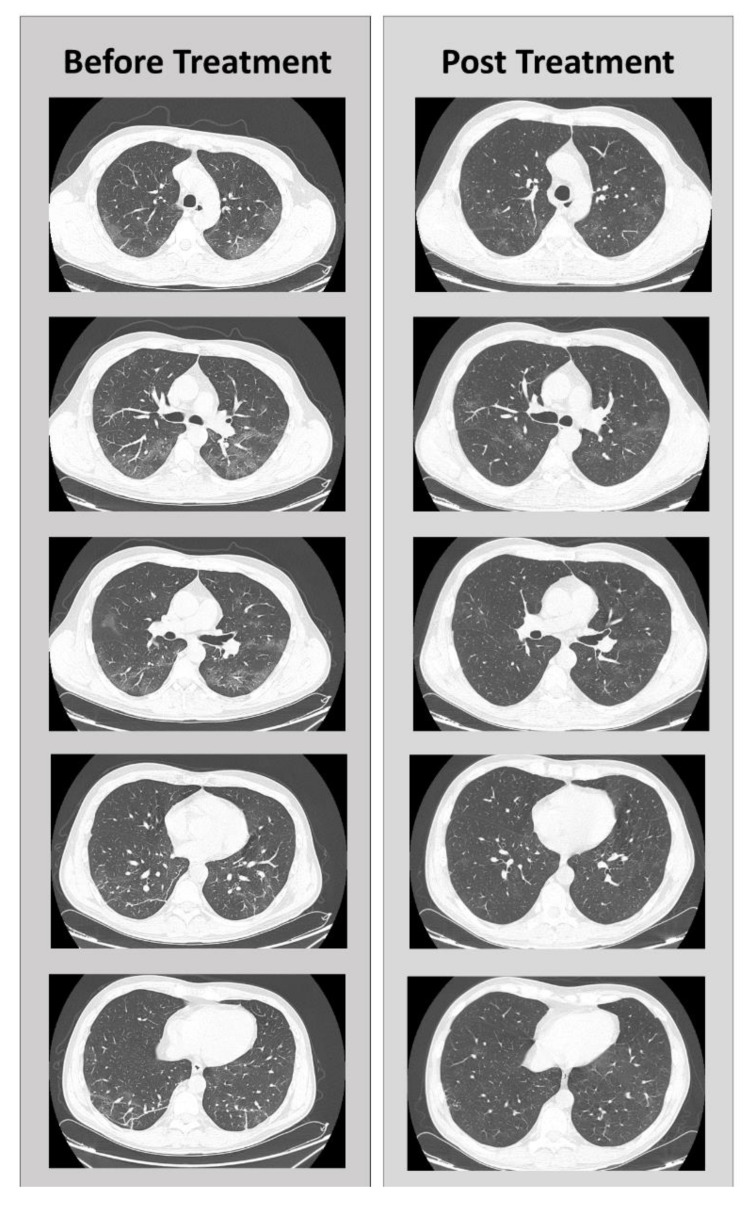
Comparison of CT scans before and after adenosine treatment, in different sections.

Finally, in the high-resolution CT-scan on April 20, a reduction in the ground-glass opacities, only appreciated in the antero-medial segment of the RUL, and a reduction of visceral pleural thickening lesions were observed. Pleuroparenchial fibrosis was no longer detectable, and there was only one residue to the right of a thin pleuroparenchymal stria with millimetric fibrotic pseudonodules. The picture appeared to be significantly improved compared with the previous CT-scans (Figure 2).

During and after treatment, adverse events related to the use of adenosine were not observed.

## 6. Conclusions

Currently inhaled adenosine is used for provocation tests, with a maximum tolerable dose of up to 40 mg. Furthermore, its therapeutic use in ARDS had already been hypothesized. For this reason, a COVID-19 patient, who showed no clinical improvement with the therapies already applied, was treated with inhaled adenosine, with a 21% oxygen mixture, due to its pathways.

In 5 days, it was possible to observe negativity of SARS-CoV2 detection and resolution of respiratory failure and radiological picture. Factors concurrent to the COVID-19 cytokine storm are several. These include obesity and its comorbidities [102,103,104,105], a pathological condition that can no longer be overlooked given the cost of human lives paid.

In conclusion, the use of adenosine can be a valid therapeutic option in COVID-19 ARDS. To overcome the compassionate use of adenosine, it is hoped that randomized controlled trials for resolution of ARDS with a cytokine storm will be launched.

## Figures and Tables

**Table 1:** Biochemical Parameters.

Parameters	Time		
	Admission	Baseline	120 h
RBC (x106/μL)	5.12	4.8	4.54
HGB (g/dL)	16.2	14.8	13.8
PLT (x103/μL)	132	301	185
WBC (x103/μL)	2.74	4.32	3.33
NEU (x103/μL)	2.14	2.93	1.85
LYMPH (x103/μL)	0,39	0.9	0.95
CRP (μg/dL)	52.4	3.14	3.14
D-DIMER (µg/L)	220	290	50

RBC: Red Blood Cells; HGB: Haemoglobin; PLT: Platelets; WBC: White Blood Cells; LYMPH: Lymphocytes; CRP: C-Reactive Protein.

## Abbreviations

ARDS	Acute respiratory distress syndrome
IL	Interleukin
TCZ	Tocilizumab
JAK	Janus kinase inhibitors
A2AR	A2A receptor
MOF	Multi organ failure
TNF-α	Tumor necrosis factor-α
hCoV	Human coronavirus
DRP	Dynamin-related proteins
PINK-1	Phosphatase and tensin homolog - induced kinase 1
RIG-I	Retinoic acid-inducible gene 1
RLRs	RIG-I-like receptors
MAVS	Mitochondrial associated antiviral signaling protein
CARDs	Caspase recruitment domains
NFkB	Nuclear factor kappa-light-chain-enhancer of activated B cells
ORF	Open reading frame
INF	Interferon
TRAF	TNF receptor associated factor
PMN	Polymorphonuclear leukocytes
ADP	Adenosine diphosphate
ATP	Adenosine triphosphate
AMP	Adenosine monophosphate
CD	Cluster of differentiation
ALI	Acute lung injury
AR	Adenosine receptors
CXCL1-3	Chemokine (C-X-C motif) ligand 3
T Regs	Regulatory T cells
PPARγ	Peroxisome proliferator-activated receptor γ
ACE2	Angiotensin-converting enzyme
EAT	Epicardial fat
GOM	Great Metropolitan Bianchi Melacrino Morelli
CT	Computed tomography
RUB	Right upper lobe
